# Exploring the Impact of Folic Acid Supplementation and Vitamin B12 Deficiency on Maternal and Fetal Outcomes in Pregnant Women with Celiac Disease

**DOI:** 10.3390/nu16183194

**Published:** 2024-09-21

**Authors:** Lily Lev, Katherine Petersen, Joseph L. Roberts, Kevin Kupferer, Steven Werder

**Affiliations:** 1 Phoenix VA Health Care System, Phoenix, AZ 85004, USA; katherine.petersen@va.gov (K.P.); joseph.roberts10@va.gov (J.L.R.); kevin.kupferer@va.gov (K.K.); steven.werder@va.gov (S.W.); 2 College of Health Solutions, Arizona State University, Phoenix, AZ 85004, USA

**Keywords:** vitamin B12, folate, folic acid, celiac disease, pregnancy

## Abstract

**Background:** Celiac disease is a chronic small intestinal immune-mediated enteropathy precipitated by exposure to dietary gluten, affecting approximately 1% of the global population and two million Americans. An increasing number of studies have identified a link between celiac disease and adverse maternal and fetal outcomes during pregnancy and after birth. Additionally, both celiac disease and pregnancy are associated with an increased risk for nutrient deficiencies, specifically vitamin B12 and folate. **Methods:** This review examines the current literature related to the folate trap and vitamin B12 deficiency in patients with celiac disease and pregnant women independently and provides rationale for future research to explore the relationship between the folate-to-12 ratio in pregnant women with celiac disease. **Results:** Deficiencies in vitamin B12 are linked with several negative maternal and fetal health outcomes including pre-eclampsia, gestational diabetes, spontaneous abortion/miscarriage, preterm birth, neural tube defects, intrauterine growth restriction, and low gestational age and birthweight. **Conclusions:** Folic acid supplementation is widely recommended during pregnancy, but complementary vitamin B12 supplementation is not standard. Physicians should consider celiac disease screening during pregnancy as well as vitamin B12 supplementation.

## 1. Introduction

**Figure 1:**
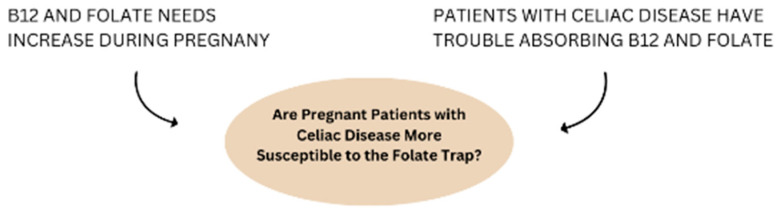
Rationale for subpopulation of interest.

Vitamin B12 and folate are essential vitamins involved in DNA synthesis, methylation pathways, and cell growth [1]. They have strong protective mental health benefits and have shown associations in chronic disease prevention [2]. However, during vitamin B12 deficiency, folate becomes ‘trapped’ in its methylated form, unable to drive other enzymatic processes essential for transmethylation pathways. This, in turn, leads to a buildup of homocysteine that has been linked to cardiovascular disease and cognitive decline, including dementia and Alzheimer’s disease [3,4]. Vitamin B12 and folate intake requirements increase during pregnancy to prevent neural tube defects, preterm birth, small gestational birthweight, and miscarriage [5,6,7]. Individuals with celiac disease (CD) are another population prone to vitamin B12 deficiency due to malabsorption [8]. Although CD is often linked to poorer pregnancy outcomes, there is limited research examining the relationship between folate and vitamin B12 deficiencies in pregnant women with celiac disease. The folate trap has the potential to exacerbate the impacts of vitamin B12 deficiency in pregnant women with CD that may be detrimental to both maternal and fetal health (Figure 1). This review explores the relationship between the folate trap and vitamin B12 deficiency in the context of pregnancy and CD, identifies the associations between CD and pregnancy outcomes, and identifies current gaps in knowledge.

## 2. Folate and Vitamin B12 Requirements and Prevalence of Deficiency

Water-soluble vitamin B12 is essential for many physiological processes that occur throughout a person’s life. Because only special microorganisms can produce vitamin B12, humans can only obtain cobalamin from food, especially foods of animal origin [9]. Deficiency has been linked with several adverse health outcomes outlined in Table 1 [10,11,12]. The Institute of Medicine recommends 2.4 μg/day of vitamin B12 for adults, with an increase to 2.6 μg/day in pregnant women [13] (Table 2). Data from the National Health and Nutrition Examination Survey (NHANES) from 2007 to 2018 found that roughly 3.6% of US adults were vitamin B12 deficient (serum < 148 pmol/L) and 12.5% had sub-clinical deficiency (<221 pmol/L) [13]. Vitamin B12 deficiency is particularly prevalent in low- and middle-income countries (40% in Latin America, 70% in Sub-Saharan Africa, and 70–80% in South Asia) [6,14].

Folate (vitamin B9) is also an essential water-soluble vitamin that plays an essential role in cell growth and division, DNA and RNA synthesis, and the maintenance of new cells [15]. Folate must be obtained through the diet or via supplementation from folic acid, the synthetic form of folate [16]. Folic acid is roughly twice as bioavailable than natural folate [17]. Deficiency can be caused by poor diet, malabsorption due to certain chronic and autoimmune diseases (e.g., celiac disease, short bowel syndrome, amyloidosis, gastric bypass), and drug or alcohol abuse. Folate deficiency can develop within weeks to months of a folate-deficient diet [12,18]. Table 3 outlines clinical manifestations of folate deficiency [12,18,19,20]. In a systematic review of 45 surveys conducted in 39 countries over 14 years, the prevalence of folate deficiency ranged from <1% to 88% [21]. This discrepancy was partly due to country income level and multiple methods being used to measure folate. Folate deficiency has become uncommon in the US due to fortification initiatives, and it is now estimated that more than one-third of the North American population consumes excess folate [22]. Adults are estimated to have approximately 1000 to 2000 μg of stored folate and need 400 μg per day to maintain these levels, while pregnant women require an increased intake of 600 μg per day [15] (Table 2). Normal serum folate levels are above 4 ng/mL, subclinical deficiency is between 3 and 4 ng/mL, and under 2 ng/mL is considered clinical deficiency [2].

## 3. Diagnosis of Folate and Vitamin B12 Deficiency

There are numerous methods to assess folate and vitamin B12 deficiency. The World Health Organization guidelines classify total serum vitamin B12 levels above 221 pmol/L as adequate, 148–221 pmol/L as low, and levels under 148 as deficient [5]. Serum vitamin B12 is the most widely used method in clinical practice and measures the total amount of vitamin B12 in the blood, of which only about 20% is metabolically active and available for cellular uptake, leading to the under-reporting of deficiency [11]. Holo-transcobalamin (holoTC) is considered a more accurate measure of vitamin B12, but it is less commonly used clinically [23]. After the consumption of vitamin B12, it binds to haptocorrin, intrinsic factor, and transcobalamin within the digestive tract. Holo-TC measures the amount of circulating vitamin B12 bound to transcobalamin that is bioavailable to cells. Plasma homocysteine and methylmalonic acid (MMA) are functional measures of vitamin B12 because both rely on vitamin B12 to be converted to methionine and succinyl-CoA, respectively. Elevated levels of either can indicate vitamin B12 insufficiency, with Hcy as a more sensitive measure, but MMA is more specific as HHcy may indicate other vitamin deficiencies [10]. Blood Hcy levels > 15 umol/L are considered elevated, whereas MMA levels >260 nmol/L are considered elevated [5].

As discussed above, serum folate levels under 2 ng/mL are considered deficient. However, as is true for vitamin B12, serum folate levels are not always accurate and do not account for unmetabolized folate or the variations in folate at different points in its metabolic cycle. Serum folate represents recent dietary intake, whereas red blood cell (RBC) folate is a more accurate long-term reflection [10]. Similar to vitamin B12, folate deficiency can be identified by elevated Hcy but normal plasma vitamin B12 and MMA levels.

## 4. Folate Trap

One-carbon metabolism is a series of metabolic reactions involving the donation of single carbon units (methyl groups) to aid in DNA, protein, and lipid biosynthesis and amino acid homeostasis [24]. The folate cycle is a component of one-carbon metabolism, working synergistically with the methionine cycle. In the folate cycle, during the conversion of 5-MTHF to THF, 5-MTHF donates a methyl group to cobalamin (vitamin B12), forming methylcobalamin, a reaction catalyzed by the enzyme methionine synthase. Methyl cobalamin then donates a methyl group to homocysteine to form methionine. Methionine is then converted into s-adenosyl methionine (SAM) and eventually s-adenosyl homocysteine (SAH) before returning to its homocysteine form. In the conversion of SAM to SAH, a methyl group is released to be used in the transmethylation pathway that drives gene regulation [10].

In the context of vitamin B12 deficiency, this cycle is fragmented as 5-MTHF cannot be converted to THF and homocysteine cannot accept a methyl group from methyl cobalamin. This causes folate to become “trapped” in its 5-MTHF form, unable to drive the multitude of downstream reactions. This prevents the conversion of homocysteine (Hcy) to methionine [25]. This leads to elevated blood Hcy levels, known as hyperhomocysteinemia (HHcy), which has been linked to dementia, stroke, recurrent early pregnancy loss, endothelial cell injury, and cardiovascular disease [11,25,26,27,28].

**Figure 2:**
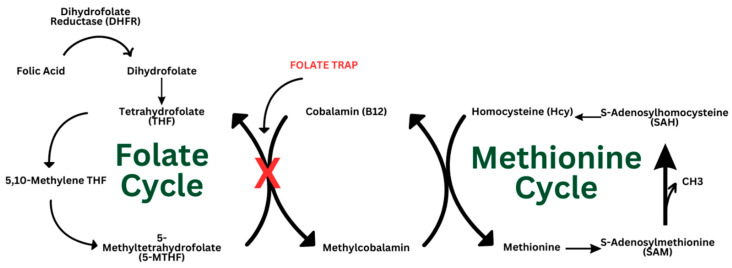
Folate and methionine metabolism. The conversion of 5-methyltetrahydrofolate (5-MTHF) to tetrahydrofolate (THF) is halted when there is a vitamin B12 deficiency. This inhibits the methionine cycle and leaves elevated homocysteine (Hcy) levels in the blood.

Humans are unable to synthesize folate naturally, so they must obtain it through dietary sources or supplements. 5-MTHF, the biologically active form of folate, is used in one-carbon metabolism and is the primary form of folate found in blood plasma. Folic acid, a synthetic monoglutamate precursor of folate, is commonly found in supplements and fortified foods, particularly grains [15]. Folic acid is then converted to its biologically active form via several multi-step enzymatic reactions. The initial step is catalyzed by dihydrofolate reductase (DHFR). Naturally occurring food folates, on the other hand, are not limited by the function of the DHFR enzyme and instead bypass this reaction when being converted into 5-MTHF (Figure 2). Regardless of the form of folate or folic acid, a vitamin B12 deficiency inhibits the folate acid cycle and halts downstream metabolic processes.

## 5. Folic Acid Fortification in the US and Exacerbation of Vitamin B12 Deficiency

### 5.1. Implementation of Folic Acid Fortification Programs

A folic acid fortification program was developed the United States in 1998, aimed toward reducing neural tube defects (NTDs). Canada, Chile, and Australia followed, and now, more than 80 countries have guidelines in place [29]. Data from the National Health and Nutrition Examination Survey (NHANES) show that, following folate fortification in the US, serum folate concentrations increased by 119–161%, and RBC folate levels rose by 44–64% [30]. The prevalence of low serum folate dropped from 21% to less than 1%, while low RBC folate declined from 38% to 5% among women of childbearing age [30]. In the US, there was a 31% decrease in NTD incidence after the initiation of the food fortification program [31]. Moreover, another randomized double-blind prevention trial revealed a 72% protective effect of folic acid supplementation on preventing NTDs [32]. Together, these findings highlight the success of folic acid fortification programs in significantly reducing the incidence of NTDs.

### 5.2. Elevated Folate Levels May Exacerbate Vitamin B12 Deficiency

Although fortification has effectively reduced folate deficiency, there are concerns that it may have introduced excessive amounts of folate into the diet. Despite the many benefits of sufficient folate levels, adequate or increased folate levels have been associated with exacerbating the health effects of vitamin B12 deficiency. High serum folate (>20 ng/mL) was found in 42% of children and 38% of elderly individuals, compared to 5% and 7% pre-fortification, respectively [30]. Furthermore, the combination of insufficient vitamin B12 and elevated serum folate increased from 0.09% to 0.61% post-fortification [33]. Elevated plasma folate levels, which have become more common post-fortification, are linked to worsening the clinical effects of vitamin B12 deficiency [34,35,36].

In addition to blood folate levels, the Framingham Offspring Cohort Study and NHANES data found an increased prevalence of high circulating folic acid after fortification [34,37]. A 2007 study in healthy adults consuming fortified bread with varying amounts of folic acid, up to 400 μg, detected unmetabolized folic acid in the plasma of those given the highest dose [38]. Similarly, unmetabolized folic acid has been found in umbilical cord blood, raising concerns that it may interfere with folate metabolism [39,40,41]. This phenomenon is thought to result from enzymatic saturation, disrupting the conversion of folic acid into 5-MTHF. These increases in blood folate and unmetabolized folic acid levels were seen after fortification guidelines were put in place, while vitamin B12 levels did not change substantially. There has been a push to include vitamin B12 fortification along with folic acid to ensure adequate levels of both nutrients and prevent exacerbation of the impacts of vitamin B12 deficiency [35,42].

## 6. Folic Acid and Vitamin B12 Needs in Pregnancy

Folic acid and vitamin B12 intake requirements increase during pregnancy to meet increased biological needs. Infant vitamin B12 and folate status at delivery is most largely influenced by maternal cobalamin and folate levels, but also varies based on placental function, gestational age, and birthweight [5,6,7]. The placenta has many folate receptors that help to regulate folate and cobalamin transfer to the fetus. Pregnancy requires a five-to-ten-fold increase in folate [15]. It is recommended that women take a folic acid supplement with at least 0.4 mg daily during pregnancy, but vitamin B12 supplementation is not recommended at the same frequency [18]. Studies have found vitamin B12 deficiencies in pregnant women to be between 18 and 43% in developed countries, with higher rates in areas with fewer resources [43,44,45,46].

Vitamin B12 deficiency and elevated Hcy concentrations are associated with the adverse fetal outcomes of spontaneous abortion/miscarriage, preterm birth, NTDs, intrauterine growth restriction, and low gestational age and birthweight [6,47,48,49,50,51,52,53,54,55,56,57,58,59,60]. This is especially prevalent when the vitamin B12-to-folate intake ratio was imbalanced (vitamin B12 < 4.0 μg per day and folate > 268 μg per day) [61]. A meta-analysis of 18 studies identified a significant relationship between vitamin B12 deficiency and low birthweight (15% increased risk) and preterm birth (21% higher risk) [62]. A recent case study followed a woman who had experienced three miscarriages and had an elevated homocysteine (Hcy) level of 15.9 μM. Despite receiving folate, vitamin B6, taurine, and cystine, her Hcy levels remained elevated, suggesting that the folate trap might have been contributing to the issue, as all other endocrine biomarkers were within normal range. She was then treated with supplemental methyl cobalamin and adenosyl cobalamin. Four months later, her Hcy dropped to within normal range at 9.9 µM, indicating that the addition of vitamin B12 was key in lowering her Hcy level [63]. Supplemental vitamin B12 and folate also significantly decreased the rate of NTDs compared to a minimal vitamin without those nutrients (13.3 vs. 22.9 per 1000) in a randomized control trial [60]. Aside from fetal outcomes, vitamin B12 deficiency also increases the risk of adverse maternal health outcomes, with up to four times the chance of preeclampsia and a significantly increased risk of insulin resistance and gestational diabetes [64,65,66].

Adequate vitamin B12 levels alone, or in conjunction with sufficient folate, can lead to positive maternal and fetal outcomes. However, vitamin B12 deficiency is often under-diagnosed in pregnant women and their children [5]. While folic acid supplementation during pregnancy is widely accepted, the addition of vitamin B12 is uncommon. This can result in imbalanced nutrient levels, potentially disrupting the methionine cycle and other downstream processes that affect the clinical health outcomes in pregnant women.

## 7. Folic Acid and Vitamin B12 Absorption in Celiac Disease

Celiac disease (CD) is defined as “a chronic small intestinal immune-mediated enteropathy precipitated by exposure to dietary gluten in genetically predisposed individuals” [67]. Gluten is a protein found in wheat, barley, rye, malt, and sometimes oats. Symptoms can present as diarrhea, weight loss, bloating, abdominal pain, and iron deficiency. The autoimmune disease can be detected through celiac-specific antibodies in bloodwork and confirmed via a duodenal mucosal biopsy [68]. CD is found in about 1% of the population, is more common in females (60–70% of CD diagnoses are women), and has increased in prevalence over the last 50 years [68,69,70,71].

CD largely impacts the villi of the small intestine, the primary site of nutrient absorption. The immune response of CD ranges in severity from mild intraepithelial lymphocytosis to total villous atrophy [8]. The disease reduces the surface area and digestive enzymes available for nutrient absorption, leading to the development of nutrient deficiencies, including iron, folic acid, B12, and B6 [68]. Folic acid is primarily absorbed in the jejunum of the small intestine, the main site impacted by CD [8]. Conversely, vitamin B12 is mostly absorbed in the ileum, the last section of the small intestine, which is also impacted in CD patients [72]. Past research has shown that 20–38% of CD patients have at least one nutritional deficiency, which could be attributed to malabsorption or deficiencies within a gluten-free diet (GFD) [8]. The GFD has been associated with the decreased intake of dietary fiber, iron, and vitamin B along with the increased consumption of caloric fats [8]. The reported prevalence of vitamin B12 deficiency specifically in patients with CD has ranged from 8 to 41% [8,73,74,75].

A complete GFD is the only treatment for the disease, with no current cure [8]. For most patients, strict adherence to a GFD will lead to the reversal of intestinal damage and eliminate symptoms over time [68]. A study of 40 patients with recent CD diagnoses followed severity of intestinal damage over a year of treatment and found that (1) the severity of the villous atrophy was linked with vitamin B12 and erythrocyte folate concentrations and (2) most biochemical markers improved within a year of treatment with a GFD [76]. However, while most serological levels return to normal with a GFD, this is not the case for all patients, and additional vitamin supplementation may be required [76,77,78,79]. However, CD is often undiagnosed in the US, which impedes a patient’s ability to access treatment [68].

In individuals diagnosed with CD, adherence to a GFD is influenced by various factors, with adherence rates ranging between 45% and 90% [80]. Barriers to diet adherence include income, knowledge about GFD, temptation control, motivation from peers, confidence in health practitioners, religion-related food constraints, the cost and availability of GF food, the amount of counseling for GFD, and contact with health care professionals [81,82]. Over 25% of subjects stated that their income could not support a GFD. A 2019 market-based study comparing gluten-free products with their glutenous counterparts found GF products to be 183% more expensive overall [83]. Thus, there are many barriers to strict adherence to a GFD which can negatively impact the overall health of an individual with CD.

## 8. Celiac Disease and Pregnancy

Several studies have identified a link between CD, especially untreated CD, and worse maternal and fetal outcomes during pregnancy. The likelihood of at least one pregnancy complication is estimated to be over 4× higher in women with CD compared to the general population [84]. A recent 18-study meta-analysis identified an increased risk for spontaneous abortion, fetal growth restriction, preterm delivery, cesarean delivery, and lower mean birthweight in women with CD [85]. A case–control study comparing treated vs. untreated CD patients found the relative risk of miscarriage to be 8.9 times higher in untreated women, and a GFD reduced that risk 9.18 times and reduced low birth rate from 29.4% to 0% [86].

Aside from pregnancy outcomes, CD also impacts fertility, with an increased likelihood for both amenorrhea and infertility [84,87,88]. A meta-analysis found three-fold increased odds of having CD in those with infertility compared to the general population [89]. Evidence suggests that with proper treatment and adherence to a GFD, many fertility and pregnancy complications associated with CD are resolved [85,86,90,91]. However, the mean age of CD diagnosis is 38 in the United States, often after fertile age in females [92]. This suggests that many women are undiagnosed during their reproductive years, as previous studies have shown that 74.5% and 85.7% of participants were diagnosed with CD only after their first pregnancy [84,93].

Asymptomatic CD is not uncommon, resulting in lack of diagnoses and/or treatment. The Mayo Clinic reported only 6% of CD patients to present with classic symptoms, while 66% presented with non-typical symptoms, and approximately 28% of patients were asymptomatic [89]. A case study of a 37-year-old woman in her third pregnancy, with one uncomplicated past pregnancy and one with intrauterine fetal death (IUFD), revealed asymptomatic CD. In her third pregnancy, the patient presented with vaginal bleeding, iron deficiency, and high IgA levels. CD was confirmed via biopsy. After treatment with iron supplementation and a strict GFD, all biomarkers resolved, and the patient gave birth to a healthy son [94]. Despite most evidence supporting the effectiveness of a GFD in reversing negative pregnancy outcomes in women with CD, lack of diagnosis or adherence to treatment remains a significant barrier for addressing these concerns.

### Celiac Disease Screening in Pregnancy

CD screening includes bloodwork to assess celiac-specific antibodies and confirmation with a duodenal mucosal biopsy [68]. As outlined in the 1968 World Health Organization principles for disease screening, it is beneficial to implement a formal screening program when early detection will improve the disease outcome [95]. Since CD can be managed through adherence to a GFD, early screening will allow patients with the disease, especially those presenting without symptoms, to resolve intestinal inflammatory markers sooner. Pregnant women are at higher risk for several negative health outcomes that celiac disease may exacerbate, making screening for the condition essential in this population.

Empirically, pregnant women aged 30 or older are recommended to be screened for gestational diabetes with an oral glucose tolerance test due to the increased risk during pregnancy [96]. Similarly, pregnant women with celiac disease are at higher risk for maternal and fetal conditions outlined earlier in the paper, which can be reversed with a GFD. Even if no formal screening program is implemented, clinicians should be aware of the relationship and should implement screening on a case-by-case basis.

## 9. Conclusions

An extensive amount of research has been conducted on the folate trap, the importance of vitamin B12 and folate supplementation in pregnancy, vitamin B12 deficiency in CD, and associations between pregnancy and CD. What has remained largely understudied are the implications of vitamin B12 deficiency in pregnant women with CD and how the folate trap may play a role in negative health outcomes. Folic acid supplementation is widely recommended during pregnancy, but recommendations for concurrent vitamin B12 supplementation are not widely practiced. Numerous studies have identified the harmful effects of vitamin B12 deficiency, which has been shown to be exacerbated in those with normal to high folate levels. This deficiency is especially prevalent in some subpopulations, notably in pregnant patients and in those with CD. An imbalanced folate-to-vitamin B12 ratio can cause several negative pregnancy outcomes including early abortion/miscarriage, preterm birth, NTDs, and low gestational age and birthweight. Importantly, many of these issues can be easily prevented with vitamin B12 supplementation. Similarly, CD is associated with higher rates of negative health outcomes that can be reversed or prevented with adherence to a GFD. However, treatment with a GFD is often delayed until after the reproductive years due to the frequent late diagnosis of CD. Additionally, the pathogenic nature of CD disrupts nutrient absorption in the small intestine, where both vitamin B12 and folate are primarily absorbed. This suggests that pregnant individuals with CD may be at an even higher risk of negative health effects from an imbalanced folate-to-vitamin B12 ratio.

Ultimately, there is an urgent need for additional research to determine the impact of the folate trap and health outcomes in pregnant women with CD. Variables impacting health outcomes may include length of CD diagnosis, adherence to GFD, family history of pregnancy complications, and nutrient supplementation. Clinically, it may be worthwhile to screen for both CD and vitamin B12 deficiency in pregnant patients to identify those who may benefit from GFD or vitamin B12 supplementation intervention. Addressing vitamin B12 deficiency and CD in pregnant patients through early screening and targeted treatments may represent a strategy to significantly reduce maternal and fetal health risks.

## Figures and Tables

**Table 1:** Manifestations of vitamin B12 deficiency.

Symptoms	Signs	Lab Findings
Fatigue	Beefy red tongue	Megaloblastic anemia
Cognitive decline	Ataxia	Anisocytosis
Upper or lower extremity paresthesia	Diminished proprioception	Poikilocytosis
Loss of balance	Diminished vibratory sense	Hyper segmented neutrophils
Falls	Romberg’s sign	Hyperhomocysteinemia

**Table 2:** Vitamin B12 and folate intake recommended dietary allowance (RDA).

	Vitamin B12	Folate
Adults	2.4 μg/day	400 μg/day
Pregnant Women	2.6 μg/day	600 μg/day

**Table 3:** Manifestations of folate deficiency.

Symptoms	Signs	Lab Findings
Fatigue	Pale skin	Megaloblastic anemia
Cognitive decline	Mouth sores	Anisocytosis
Irritability	Diminished proprioception	Poikilocytosis
Decreased Appetite	Diminished vibratory sense	Hyper segmented neutrophils
Diarrhea	Smooth and tender tongue	Hyperhomocysteinemia
